# Effects of exercise and diet-induced weight loss on markers of inflammation II: impact on microRNA 21 and microRNA 146a expression and their regulatory role

**DOI:** 10.1186/1550-2783-10-S1-P24

**Published:** 2013-12-06

**Authors:** S Simbo, A Roque-Andrade, B Lockard, C Baetge, S Mertens-Talcott, C Rasmussen, R Kreider

**Affiliations:** 1 Exercise & Sport Nutrition Lab, Department of Health and Kinesiology, Texas A&M University, College Station, TX 77843, USA; 2 Department of Nutrition and Food Science, Texas A&M University, College Station, TX 77843, USA

## Background

Obesity has been associated with inflammation. However, the mechanisms are not well understood. The purpose of this study was to determine if exercise and diet-induced weight loss would affect markers of inflammation via the Phosphatase and Tensin homologue Deleted from Chromosome-10 (PTEN), TNF receptor-associated factor 6 (TRAF6), Phosphatidylinositol-3-kinase (PI3k), Protein Kinase B (AKT or PKB), Nuclear Factor kappa Beta (NF-kB) signaling pathway through the regulation of microRNA 21 and microRNA 146a expression.

## Methods

Forty-five overweight and sedentary women (48.16±10.5 yr, 45.9±4.4% body fat, BMI 35.6±5.6 kg/m^2^) were randomized into a control group (C, n=18) or an exercise and diet-induced weight loss group (EX, n=27). Participants followed an energy-restricted diet (1,200 kcal/d for 1 week and 1,500 kcal/d for 11weeks; 30% CHO, 45% P, and 25% F) while participating in a circuit resistance-training (3d/wk) program. The resistance training program included 30 seconds of resistance exercise interspersed with 30 seconds of continuous movement (calisthenics). Whole blood samples were obtained at 0 and 12 wks and centrifuged immediately to obtain white blood cells buffy coat for mRNA isolation. The microRNA (21 and 146a) and mRNA of IL-6, TNF-α, (PTEN, TRAF6)/PI3k/AKT/NF-kB signaling pathway expression levels were measured in serum/WBC (buffy coat) by real-time RT-PCR and normalized using ΔΔCt formula with U6B as a normalization control for the microRNAs and Glyceraldehyde-3-phosphate dehydrogenase (GAPDH) as an endogenous control for mRNAs. The ΔΔ C Baetge, B Lockard Ct formula, Ct represents the real time cycle number at which microRNA and mRNA probe fluorescence is exponential. Data were analyzed by MANOVA and presented as changes from baseline after 12 wks.

## Results

An overall significant MANOVA interaction was observed among EX and C groups (Wilks’ Lambda p<0.001). MANOVA univariate analysis revealed no significant interactions among groups in changes in microRNA 146a (EX -0.73±2.0; C -0.28±2.1, p=0.46); TRAF6 (EX –1.35±2.7; C -0.74±3.5, p=0.52); mRNA expression levels of PI3K (EX -2.4±4.5; C -1.8±2.9, p=0.66); AKT (EX -1.34±4.2; C -0.67±7.4, p=0.70); or, mRNA NF-kB (EX -1.6±3.2; C -0.73±3.2, p=0.40). Significant interactions were observed among groups in changes in microRNA 21 (EX -1.5±2.34; C 0.13±2.2, p=0.03); mRNA expression level of its target gene PTEN (EX -4.5±3.2; C -1.6±3.4, p=0.005); mRNA IL-6 (EX -2.8±3.6; C 2.8±2.2, p<0.001); and, mRNA TNF-α expression levels (EX -0.52±2.5; C 2.3±1.9, p<0.001). Exercise and diet-induced changes in mRNA IL-6 and mRNA TNF-α expression were positively and significantly correlated to changes in body weight (r=0.47, r=0.30), fat mass (r=0.48, r=0.31), and percent body fat (r=0.48, r=0.32), respectively.

## Conclusion

Results of this study indicate that exercise and diet-induced weight loss affects molecular changes in circulating microRNAs, significantly affects microRNA 21 and its target gene PTEN, mRNA TNF-α, and mRNA IL-6 levels suggesting a anti-inflammatory response compared to a control group. These findings suggest that exercise and diet-induced weight loss is significantly associated with a reduction in inflammation. However, more research is needed to understand microRNA regulation associated with inflammation in response to exercise.

